# Climate-induced vulnerability and health outcomes in urban Bangladesh: the mediating role of psychological stress

**DOI:** 10.3389/fpubh.2026.1860955

**Published:** 2026-06-11

**Authors:** Yu Xunda, Konstantin Moskalenko, Mohammad Anisur Rahaman, Natalia Taranova, Md. Rakibul Islam

**Affiliations:** 1 Department of Government Administration, School of Public Affairs, Zhejiang University, Hangzhou, China; 2 Department of Sociology, Gopalganj Science and Technology University, Gopalganj, Bangladesh; 3 Department of International Scientific Cooperation, Saint Petersburg State Pediatric Medical University, Saint Petersburg, Russia

**Keywords:** climate change and health, climate vulnerability, informal settlements, psychological stress, urban health

## Abstract

**Background:**

Climate change poses growing risks to urban health, particularly in low-income informal settlements where exposure to environmental hazards is frequent and adaptive capacity is limited. However, the psychosocial mechanisms linking climate exposure to health outcomes remain underexplored.

**Objective:**

This study examines the relationship between climate exposure, psychological stress, and physical health outcomes, with a focus on the mediating role of stress among low-income urban populations in Dhaka, Bangladesh.

**Methods:**

A cross-sectional quantitative study was conducted using household survey data (*n =* 384), complemented by secondary environmental data. Multivariable regression, mediation analysis with bootstrapping, and Structural Equation Modeling (SEM) were employed to assess direct and indirect pathways.

**Results:**

Climate exposures, including heatwaves, flooding, and waterlogging, were highly prevalent and significantly associated with increased psychological stress. Regression and mediation analyses indicate that psychological stress partially mediates the relationship between climate vulnerability and adverse health outcomes, while direct effects remain significant. Socioeconomic disadvantage and poor housing conditions further amplify health risks.

**Conclusion:**

Climate change functions as both an environmental and psychosocial stressor in low-income urban settings. Integrated interventions addressing environmental exposure, mental health, and structural inequalities are essential to mitigate climate-related health risks.

## 1 Introduction

### 1.1 Climate change and urban vulnerability in the Global South

Climate change is intensifying the frequency and severity of extreme weather events such as heatwaves, flooding, and waterlogging, posing significant risks to urban populations worldwide (1). Climate change in the contemporary era is increasingly understood within the framework of the Anthropocene, a period characterized by large-scale human alteration of the Earth’s climate systems through fossil fuel combustion, carbon-intensive urbanization, industrial production, and extractivist exploitation of natural resources (2). Unlike long-term natural climatic variability, current patterns of extreme heat, flooding, hydrological instability, and environmental disruption are strongly associated with anthropogenic greenhouse gas emissions and unequal models of economic development rooted in resource extraction and unsustainable urban expansion (3). These climate events also include prolonged droughts, sea-level rise, saline intrusion, and intensified hydrological extremes, which are increasingly linked to fossil fuel-driven warming, accelerated ice-sheet loss, and human-induced transformations of regional and global climate systems. Recent evidence demonstrates that rapidly urbanizing regions of the Global South experience disproportionate exposure to these anthropogenically intensified climate hazards due to historically uneven infrastructure development, ecological degradation, and structural inequality (4). These risks are particularly pronounced in the Global South, where rapid urbanization, socioeconomic inequality, and inadequate infrastructure interact to amplify vulnerability (5, 6). Urban informal settlements, characterized by high population density, poor housing conditions, and limited access to basic services, are disproportionately exposed to climate-related hazards (7). In such contexts, environmental stressors intersect with structural disadvantages, creating compounded vulnerabilities that extend beyond physical infrastructure to affect human health and wellbeing (8, 9). In cities such as Dhaka, climate-related risks therefore emerge not simply as natural environmental phenomena, but as socially and historically produced vulnerabilities embedded within broader political-economic and urban development processes.

### 1.2 Climate exposure as a psychosocial stressor

Emerging evidence suggests that climate-related exposures are not only environmental threats but also significant psychosocial stressors. Individuals exposed to recurrent climate events—including heat stress, flooding, and livelihood disruption—experience elevated levels of psychological distress, including anxiety, depression, and chronic stress (10, 11). These stress responses are often prolonged and cumulative, contributing to sustained psychosocial burdens rather than short-term reactions. Chronic stress triggered by environmental instability can induce physiological changes, including impaired immune functioning, increased cardiovascular risk, and metabolic dysregulation (12, 13). As such, climate vulnerability operates as a multidimensional risk factor that simultaneously affects environmental, psychological, and biological systems.

### 1.3 Research gaps in climate–health linkages

A growing body of research from the Global South has documented the health consequences of extreme climate events, particularly among populations exposed to recurrent flooding, heat stress, displacement, food insecurity, and environmental degradation (8, 14). Recent studies have also demonstrated that climate-related risks disproportionately affect low-income urban communities where inadequate housing, insecure livelihoods, and limited access to health services intensify vulnerability (7, 15). However, much of the existing evidence remains analytically fragmented, often examining environmental exposure, mental health outcomes, and physical illness separately rather than within an integrated framework of health vulnerability and social determinants of health. In particular, limited research has simultaneously examined how chronic climate exposures interact with psychological stress, socioeconomic inequality, and structural urban conditions to shape multidimensional health outcomes in informal urban settlements. Strengthening this integrative and pathway-based evidence is especially important in rapidly urbanizing and climate-vulnerable settings such as Dhaka, where environmental risks and structural disadvantage intersect in complex ways.

While prior studies have examined climate exposure, mental health, or physical illness separately, this study advances existing knowledge by integrating environmental exposure, psychological stress, and physical health outcomes within a single empirical framework in a low-income urban context. The study further contributes by examining psychological stress as a mediating pathway linking climate vulnerability and health outcomes among residents of informal settlements in Dhaka, Bangladesh. By combining climate vulnerability indicators, psychosocial measures, and pathway-based quantitative analysis, the study provides context-specific evidence on multidimensional climate–health relationships in rapidly urbanizing settings of the Global South. Strengthening this integrative and pathway-based evidence is especially important in rapidly urbanizing and climate-vulnerable settings such as Dhaka, where environmental risks and structural disadvantage intersect in complex ways (8, 15).

### 1.4 Urban climate vulnerability in Dhaka

Dhaka, one of the fastest-growing megacities globally, provides a critical context for examining these dynamics. Rapid urban expansion, coupled with inadequate planning and infrastructure, has resulted in the proliferation of informal settlements in environmentally vulnerable areas (16, 17). These settlements are frequently exposed to flooding, prolonged waterlogging, and extreme heat, while residents face limited adaptive capacity due to poverty, insecure livelihoods, and restricted access to services (18, 19). GIS-based assessments indicate that a substantial proportion of Dhaka is highly vulnerable to waterlogging, with informal settlements disproportionately affected (20). Additionally, poor housing materials, inadequate ventilation, and lack of cooling mechanisms significantly intensify heat stress among residents (21). These intersecting environmental and socioeconomic conditions create a setting in which climate exposure, psychological stress, and health outcomes are deeply interconnected. The recurrent and seasonal nature of heatwaves, monsoon flooding, and prolonged waterlogging in Dhaka further makes the city an analytically important setting for examining chronic climate-related psychosocial and health vulnerabilities among low-income urban populations.

### 1.5 Study objectives and research questions

The primary objective of this study is to examine how climate-induced vulnerability influences psychological stress and physical health outcomes among low-income urban populations in Dhaka, Bangladesh. Specifically, the study addresses the following objectives:

1. assess the extent of climate exposure and vulnerability in informal settlements;
2. analyze the relationship between climate-related exposures and psychological stress; and
3. evaluate the mediating role of psychological stress in linking climate vulnerability to physical health outcomes.

Accordingly, the study addresses the following research questions:

1. To what extent are residents of informal settlements exposed to climate-related vulnerability and environmental stressors?
2. What is the relationship between climate-related exposures and psychological stress among low-income urban populations in Dhaka?
3. To what extent does psychological stress mediate the relationship between climate vulnerability and physical health outcomes among low-income urban populations in Dhaka?

## 2 Conceptual and theoretical framework

### 2.1 Climate-induced vulnerability framework

This study adopts a climate-induced vulnerability framework that conceptualizes vulnerability as a function of exposure, sensitivity, and adaptive capacity, a model widely applied in climate and urban health research (6, 8). Exposure refers to the extent to which individuals and communities experience climate-related stressors such as heatwaves, flooding, and waterlogging. Sensitivity captures the degree to which social, economic, and biological conditions increase susceptibility to harm, while adaptive capacity reflects the ability to anticipate, cope with, and recover from climate impacts.

In low-income urban contexts of the Global South, these components interact in structurally unequal ways. High exposure to environmental hazards is often compounded by elevated sensitivity arising from precarious housing, informal employment, and limited access to basic services (7, 18). At the same time, adaptive capacity remains constrained by poverty, weak governance, and infrastructural deficits (16, 22). Empirical studies further demonstrate that inadequate urban infrastructure and poor environmental management intensify vulnerability to flooding and waterlogging in rapidly urbanizing cities (19, 20). This framework provides a multidimensional lens for understanding how climate risks are socially produced and unevenly distributed across urban populations.

### 2.2 Stress process theory and environmental stress models

To explain how climate vulnerability translates into health outcomes, this study draws on Stress Process Theory and environmental stress models. Stress Process Theory conceptualizes stress as a socially structured process in which exposure to chronic stressors rooted in inequality leads to psychological distress and subsequent health deterioration (23). Key mechanisms include stress proliferation, cumulative exposure, and unequal access to coping resources. Environmental stress models complement this perspective by emphasizing the role of physical environmental conditions such as extreme heat, crowding, pollution, and degraded living environments as persistent stressors that exceed individuals’ coping capacities (24). In climate-vulnerable urban settings, recurrent environmental shocks such as flooding and heatwaves intensify stress exposure and amplify psychosocial burdens (10). Together, these frameworks explain how environmental hazards are internalized as psychosocial stress, producing cumulative psychological and physiological strain.

### 2.3 Social determinants of health framework

The Social Determinants of Health (SDH) framework situates health outcomes within broader structural and socioeconomic conditions, emphasizing that health is shaped by the environments in which people are born, live, work, and age (25). Recent scholarship highlights climate change as a structural determinant of health that amplifies existing inequalities by disproportionately affecting populations with limited economic resources, insecure housing, and restricted healthcare access (15). In low-income urban communities, climate stressors interact with determinants such as income, education, gender, and neighborhood infrastructure, producing compounded vulnerabilities. Gendered dimensions of vulnerability further intensify health risks, particularly among women who often face limited access to adaptive resources and increased caregiving burdens (24). These intersecting disadvantages not only increase exposure and sensitivity to climate risks but also limit adaptive capacity and coping mechanisms. Integrating the SDH framework enables this study to foreground the structural drivers of climate-related health inequalities.

### 2.4 Integrated conceptual pathway: linking climate vulnerability, psychological stress, and health outcomes

Drawing on these theoretical perspectives, this study proposes an integrated conceptual pathway linking climate-induced vulnerability to health outcomes through psychological stress. Climate change is conceptualized as a distal structural driver that generates proximal environmental and social stressors, including heat exposure, flooding, livelihood disruption, and service insecurity (1, 8). These stressors elevate levels of psychological distress, which in turn influence both mental and physical health outcomes. Psychological stress is positioned as a central mediating mechanism, consistent with Stress Process Theory, through which environmental exposures are translated into adverse health effects. Chronic psychological stress influence to physiological dysregulation through neuroendocrine, immune, and behavioral pathways, potentially increasing susceptibility to certain adverse health outcomes, particularly cardiovascular and stress-sensitive chronic conditions (26, 27). However, the relationship between psychological stress and climate-related physical illnesses is complex and potentially bidirectional. In the context of heat-related illness and infectious diseases, environmental exposure itself remains a primary etiological driver, while psychological stress may exacerbate vulnerability by influencing immune function, sleep, hydration practices, healthcare-seeking behavior, and adaptive coping capacity rather than acting as an independent direct cause (8, 11). Similarly, pre-existing chronic illnesses may also increase psychological distress, particularly in climate-vulnerable settings where recurrent environmental disruption limits disease management and access to care. Therefore, the mediation pathways examined in this study should be interpreted as probabilistic psychosocial mechanisms within a broader biopsychosocial and environmental risk framework, rather than strictly unidirectional causal relationships (12). Empirical evidence supports these mediated pathways, highlighting psychological stress as a critical link between climate exposure and health outcomes, particularly in vulnerable populations (10). Importantly, these climate–health pathways are socially stratified rather than uniformly distributed across populations. Structural determinants of health including poverty, insecure housing, labor precarity, unequal urban infrastructure, and limited access to health and social protection systems shape differential exposure and vulnerability to climate-related stressors (25). Within these unequal structural conditions, socially patterned characteristics such as gender, educational attainment, age, and pre-existing health conditions influence how individuals experience, respond to, and cope with climate-related psychological and physical health risks. In low-income urban settlements, these intersecting social and structural inequalities contribute to cumulative vulnerability and uneven health outcomes under conditions of recurrent climate stress. Individuals with fewer resources are more likely to experience intensified stress responses and greater health impacts, reflecting cumulative vulnerability processes.

**Figure 1:**
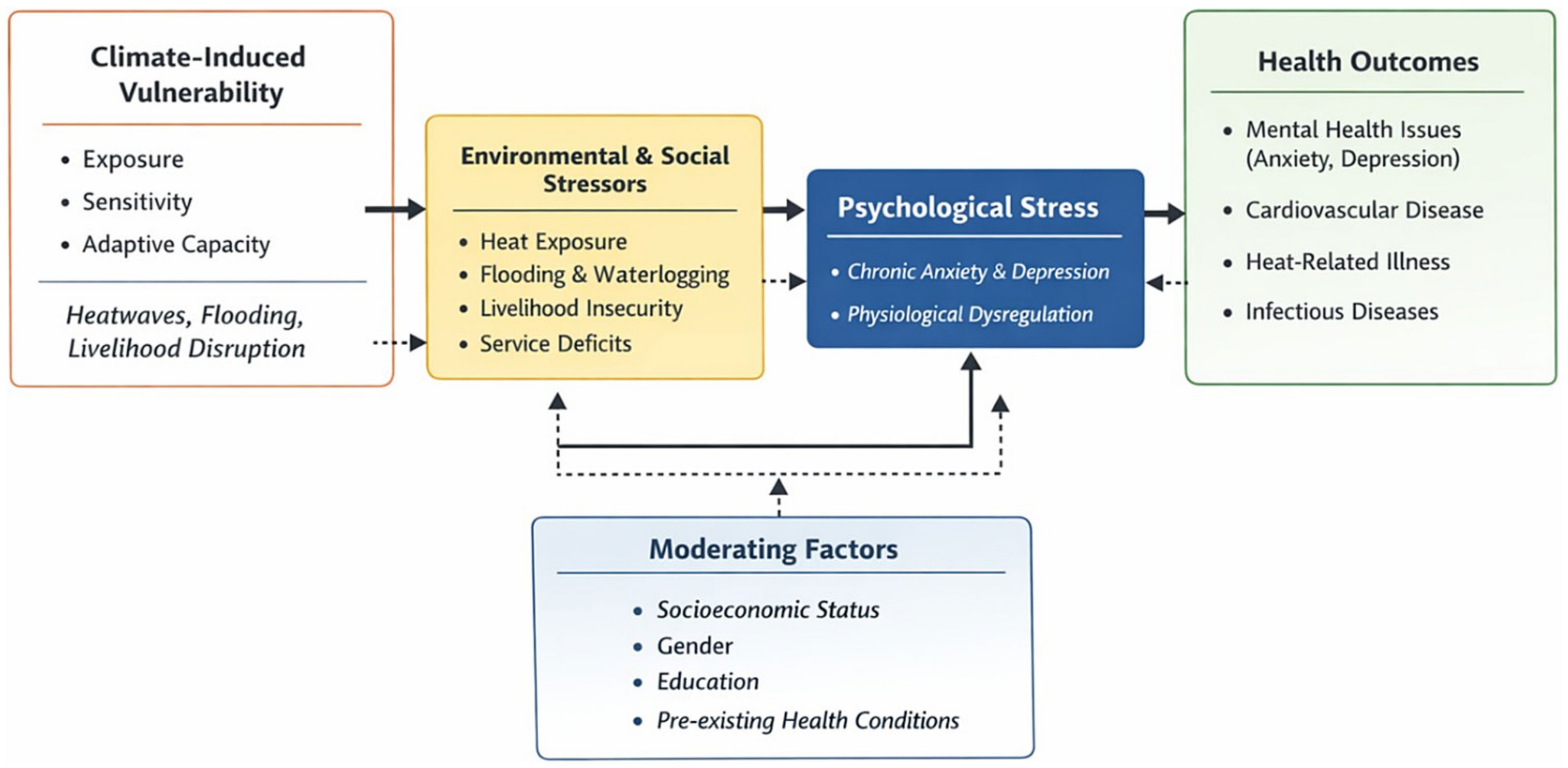
Conceptual framework.

This integrated framework directly informs the study’s empirical design and analytical approach. First, climate-induced vulnerability is operationalized through exposure, sensitivity, and adaptive capacity indicators, aligning with the objective of assessing the extent of climate vulnerability in informal settlements. Second, psychological stress is modeled as a mediating variable linking climate exposure to health outcomes, addressing the objective of examining psychosocial pathways. Third, socioeconomic and demographic factors are incorporated as moderating variables, reflecting the role of structural inequalities in shaping these relationships. Accordingly, the framework guides the use of multivariable regression, mediation analysis, and structural equation modeling (SEM) to test direct effects (from climate exposure to health outcomes), indirect effects (from climate exposure to psychological stress to health outcomes), and moderation effects across different population groups. By integrating mediation and moderation within a single analytical structure, the framework advances a multidimensional understanding of climate-health linkages in low-income urban settings, as shown in Figure 1.

## 3 Methods

### 3.1 Study design

A quantitative cross-sectional design was employed to examine the relationships between climate-induced vulnerability, psychological stress, and physical health outcomes among residents of low-income urban communities in Dhaka, Bangladesh. The study was based on a structured household survey (*n =* 384) supplemented by secondary climate and environmental data. This approach was selected to enable statistical estimation of direct and indirect associations among variables and to test theoretically grounded mediation pathways derived from Stress Process Theory and environmental stress models. Data collection was employed over a four-month period from May 2025 to August 2025. This design facilitates systematic measurement, comparability, and generalizability within the study population. Given the study’s objective to examine the mediating role of psychological stress in the relationship between climate vulnerability and health outcomes, regression-based mediation analysis and Structural Equation Modeling (SEM) were employed. These techniques allow simultaneous estimation of multiple pathways, adjustment for confounding variables, and evaluation of model fit, thereby strengthening internal validity and theoretical alignment. However, given the cross-sectional nature of the data, the identified associations and mediation pathways should be interpreted as indicative rather than strictly causal.

### 3.2 Study area and sampling

The study was conducted in selected informal settlements within the Dhaka North City Corporation (DNCC) and Dhaka South City Corporation (DSCC), Bangladesh. This area was purposively selected due to its representation of one of the most climate-vulnerable and densely populated megacities in the Global South, where rapid urbanization, environmental stress, and socioeconomic inequality intersect. According to the Bangladesh Bureau of Statistics (28), the Dhaka Metropolitan Area accommodates more than 22 million residents, with extremely high population density and substantial concentrations of low-income households living in informal settlements. Recent urban assessments further indicate that a significant proportion of Dhaka’s residents live in climate-exposed settlements characterized by overcrowded housing, inadequate drainage systems, insecure tenure, limited sanitation coverage, and restricted access to basic services (16, 18). These structural conditions increase sensitivity to climate-related hazards and reduce adaptive capacity among low-income urban populations.

Dhaka was also selected because of its recurrent exposure to climate-related extreme events, particularly heatwaves, monsoon flooding, and prolonged urban waterlogging. Bangladesh Meteorological Department reports and recent urban climate studies indicate that Dhaka has experienced increasing frequency and intensity of extreme heat events over the last decade, especially during the pre-monsoon season (29), with recurrent episodes of temperatures exceeding seasonal norms (8). Similarly, monsoon flooding and waterlogging occur almost annually in several low-lying and poorly drained parts of the city, often disrupting housing, sanitation, mobility, livelihoods, and access to safe water for extended periods (19). GIS-based analyses further demonstrate that informal settlements located near drainage canals, wetlands, and low-elevation zones are disproportionately exposed to recurrent flooding and prolonged water stagnation (18). These environmental and structural characteristics make Dhaka an appropriate and analytically important setting for examining the interrelationships among climate exposure, psychological stress, and health outcomes in low-income urban communities.

The sample size of 384 households was determined using Cochran’s sample size formula for large populations at a 95% confidence level and 5% margin of error, assuming maximum variability (*p* = 0.5). Dhaka Metropolitan Area contains several million households distributed across densely populated urban settlements (28), making the use of Cochran’s formula appropriate for estimating a representative sample in the absence of a complete household sampling frame for informal settlements. The calculated minimum sample size was 384 households, which was considered sufficient to ensure statistical reliability and representativeness for the study objectives. Given the absence of a comprehensive household sampling frame for informal settlements, a multistage stratified random sampling strategy was employed. First, informal settlements were stratified based on flood vulnerability levels (high vs. moderate) using secondary GIS and municipal data. Second, within each selected settlement cluster, households were selected using systematic random sampling. A sampling interval (k) was calculated by dividing the estimated number of households in each cluster by the allocated sample size. The first household was selected randomly, followed by every kth household thereafter. To minimize selection bias, standardized household listing procedures were followed within each cluster. Non-response was defined as: (a) repeated absence of eligible respondents from the selected household after two revisits at different times of the day, (b) refusal to participate, (c) inability of the respondent to provide informed responses due to serious illness or communication difficulties, (d) incomplete questionnaires with substantial missing data. In such cases, replacement households were selected using the same systematic sampling interval within the same settlement cluster to maintain sampling consistency and reduce potential selection bias.

### 3.3 Data collection procedures

Data collection was conducted over a four-month period from May 2025 to August 2025, capturing both peak pre-monsoon heat exposure and monsoon flooding conditions. A structured questionnaire was developed, pretested, and refined to ensure clarity, cultural appropriateness, and contextual relevance. Trained field enumerators conducted face-to-face interviews using standardized survey instruments. Field supervision and periodic spot checks were implemented to ensure data quality and minimize interviewer bias. Psychological stress was measured using validated instruments, including the Perceived Stress Scale (PSS-10) and the Depression, Anxiety, and Stress Scale (DASS-21). Reliability was assessed using Cronbach’s alpha, with coefficients exceeding 0.70, indicating satisfactory internal consistency. Construct validity was supported through consistency with previously validated Bangla adaptations. Secondary environmental data on temperature, rainfall, flooding, and waterlogging were obtained from the Bangladesh Meteorological Department and the Dhaka Water Supply and Sewerage Authority to validate self-reported exposure measures and contextualize neighborhood-level vulnerability patterns. Informed consent was obtained from all participants prior to data collection. Participation was voluntary, confidentiality was maintained, and respondents were informed of their right to withdraw at any stage.

### 3.4 Measures and variables

The structured questionnaire consisted of five major domains: (1) socio-demographic and household characteristics, (2) climate-related exposure and vulnerability indicators, (3) psychological stress measures, (4) physical health outcomes, and (5) adaptive capacity and service access variables. The selection of variables was informed by prior climate–health and social determinants of health literature (8, 15).

#### 3.4.1 Socio-demographic and household variables

Socio-demographic variables included age, gender, educational attainment, occupation, monthly household income, household size, housing type, access to safe drinking water, sanitation condition, neighborhood population density, and drainage/flood-risk conditions. In addition, household service-related indicators were included to capture environmental and infrastructural conditions associated with climate vulnerability. Variables such as access to safe drinking water, sanitation facilities, and neighborhood drainage conditions were incorporated because previous evidence suggests that disruption of these services during heatwaves, flooding, and waterlogging directly affects exposure pathways and health risks in informal urban settlements. Household access to electricity and alternative energy sources (e.g., solar systems) was not included as a study variable because the present analytical framework primarily focused on climate-sensitive environmental exposures and basic service disruptions directly linked to waterborne risk, sanitation failure, and flood-related health impacts. Moreover, preliminary field assessments indicated relatively limited variability in household electricity access across the selected settlements, reducing its expected explanatory contribution within the climate vulnerability framework applied in this study. Age was measured as a categorical variable (18–29, 30–44, 45–59, and ≥60 years). Gender was coded as male = 0 and female = 1. Educational attainment was categorized as no formal education, primary education, and secondary or higher education. Monthly household income was measured in Bangladeshi Taka (BDT) and grouped into three categories (<10,000; 10,000–19,999; and ≥20,000 BDT). Housing condition variables included housing materials, overcrowding, ventilation status, sanitation facilities, and access to safe water. These socio-demographic and household variables were included as covariates and moderating factors in the multivariable regression and Structural Equation Modeling (SEM) analyses because previous research demonstrates their association with climate vulnerability, psychological stress, and health inequalities (7, 25).

#### 3.4.2 Climate vulnerability variables

Climate-induced vulnerability was operationalized using three dimensions: exposure, sensitivity, and adaptive capacity (30). Exposure indicators included frequency of heatwaves, flooding, and prolonged waterlogging during the previous 12 months. Sensitivity indicators included poor housing quality, overcrowding, inadequate ventilation, sanitation disruption, and climate-sensitive livelihood disruption. Adaptive capacity variables included household income, access to services, preparedness support, and safe water availability during climate events. A composite Climate Vulnerability Index (CVI) was constructed by standardizing and aggregating exposure, sensitivity, and adaptive capacity indicators using z-scores. Higher CVI values represented greater climate vulnerability. Although household energy availability may influence adaptive responses to extreme heat and environmental stress, energy-related indicators were not incorporated into the Climate Vulnerability Index (CVI) because the index was designed to prioritize climate-sensitive exposures and service disruptions with direct relevance to recurrent flooding, waterlogging, sanitation interruption, and safe water access in informal settlements. This decision was made to maintain conceptual consistency with the study objectives and avoid introducing variables not systematically measured across all sampled households.

#### 3.4.3 Psychological stress variables

Psychological stress was measured using the Perceived Stress Scale (PSS-10) (26), and selected dimensions of the Depression, Anxiety, and Stress Scale (DASS-21) (31). The instruments were translated, adapted, and pretested for contextual suitability in Bangla-speaking urban communities. PSS-10 scores were treated as continuous variables, with higher scores indicating greater perceived psychological stress. Internal consistency reliability exceeded the recommended threshold (Cronbach’s *α* > 0.70). Psychological stress functioned as the primary mediating variable in the analytical framework linking climate vulnerability and physical health outcomes.

#### 3.4.4 Physical health outcome variables

Physical health outcome variables included heat-related illness, waterborne and vector-borne infectious diseases, chronic disease exacerbation (e.g., hypertension, respiratory illness, diabetes), and nutritional insecurity. Health outcomes were assessed using self-reported symptoms, reported illness experiences during climate events, and available household medical documentation where accessible. For logistic regression analyses, binary variables were created (1 = presence of adverse health outcome; 0 = absence). In SEM analysis, a composite physical health outcome index was constructed using standardized indicators of climate-sensitive health conditions.

#### 3.4.5 Analytical role of variables

In the analytical framework, climate vulnerability variables served as the principal independent variables, psychological stress served as the mediating variable, and physical health outcomes served as the dependent variables. Socio-demographic and household characteristics were incorporated as control and moderating variables in multivariable regression, mediation analysis, and SEM models.

### 3.5 Analytical strategy

Data analysis was conducted using SPSS 26 version. Descriptive statistics (means, standard deviations, and frequencies) were used to summarize key variables. Bivariate analyses (*t*-tests and chi-square tests) examined associations between climate exposure indicators and psychological stress. Multivariable linear regression models were used to estimate the effects of climate exposure on psychological stress, adjusting for socioeconomic and demographic covariates. Moreover, Binary logistic regression models were employed to assess the likelihood of adverse physical health outcomes, with results reported as adjusted odds ratios (ORs) and 95% confidence intervals. Mediation analysis was conducted using a regression-based approach with bootstrapping (5,000 resamples) to estimate indirect effects and assess the mediating role of psychological stress (32). Structural Equation Modeling (SEM) was performed to simultaneously estimate direct and indirect pathways among climate exposure, psychological stress, and health outcomes. Model fit was evaluated using standard indices, including the Comparative Fit Index (CFI), Tucker–Lewis Index (TLI), Root Mean Square Error of Approximation (RMSEA), and Standardized Root Mean Square Residual (SRMR).

### 3.6 Ethical considerations

The study adhered to international research ethics standards. Participants were informed about the study’s purpose, procedures, and potential risks, and provided written or verbal informed consent prior to participation. Personal identifiers were removed, and data were stored securely with restricted access. The study protocol was approved by the Institutional Review Board (IRB) of Gopalganj Science and Technology University (GSTU), Bangladesh and followed the principles of the Declaration of Helsinki (33).

## 4 Results

To facilitate interpretation of the study findings, the results are presented using multiple complementary analytical approaches addressing the study objectives. Descriptive findings first characterize participant and climate vulnerability conditions, followed by analyses examining associations between climate exposure and psychological stress, and finally mediation and pathway analyses exploring the role of psychological stress in health outcomes. Together, these analyses provide integrated evidence addressing the study objectives from different statistical perspectives.

### 4.1 Socio-demographic characteristics of participants

Table 1 presents the socio-demographic and household characteristics of respondents living in informal settlements in Dhaka. The sample is predominantly composed of working-age individuals, with 67.2% aged between 18 and 44 years. Gender distribution is relatively balanced (51.6% male and 48.4% female). Educational attainment is low, with more than half of respondents having no formal or only primary education. Economic vulnerability is pronounced, as over 70% of households report a monthly income below BDT 20,000, and nearly half are engaged in informal or daily wage employment. Household and environmental conditions further reflect structural disadvantage. A large proportion of respondents live in kutcha or semi-pucca housing (75.5%), with high levels of overcrowding and limited access to basic services. Approximately one-third of households lack access to safe drinking water, and nearly two-thirds rely on unimproved sanitation facilities. The inclusion of water and sanitation indicators reflects the study’s emphasis on climate-sensitive service disruptions frequently affected during flooding and prolonged waterlogging events in Dhaka’s informal settlements. Additionally, 56.8% of households are located in areas with poor drainage or high flood risk, and the majority reside in densely populated neighborhoods. The descriptive profile indicates overlapping socioeconomic and infrastructural vulnerabilities, providing an important context for subsequent analyses of climate exposure, psychological stress, and health outcomes.

**Table 1:** Socio-demographic characteristics of participants.

Variable	Category	*n* (%)
Age (years)	18–29	112 (29.2%)
	30–44	146 (38.0%)
	45–59	84 (21.9%)
	≥60	42 (10.9%)
Gender	Male	198 (51.6%)
	Female	186 (48.4%)
Education	No formal education	70 (18.2%)
	Primary	140 (36.5%)
	Secondary or higher	174 (45.3%)
Household income (BDT/month)	<10,000	118 (30.7%)
	10,000–19,999	156 (40.6%)
	≥20,000	110 (28.7%)
Occupation	Unemployed	78 (20.3%)
	Daily wage/informal	184 (47.9%)
	Formal job	122 (31.8%)
Household size	1–3 members	96 (25.0%)
	4–5 members	174 (45.3%)
	≥6 members	114 (29.7%)
Housing type	Kutcha/Semi-pucca	290 (75.5%)
	Pucca	94 (24.5%)
Access to safe water	Yes	258 (67.2%)
	No	126 (32.8%)
Sanitation	Improved	140 (36.5%)
	Unimproved	244 (63.5%)
Drainage/flood risk	Poor/High risk	218 (56.8%)
	Adequate/Low risk	166 (43.2%)
Neighborhood density	High (>75,000 persons/km^2^)	226 (58.9%)
	Moderate	104 (27.1%)
	Low	54 (14.0%)

Source: Field survey, 2025.

### 4.2 Climate exposure and climate-induced vulnerability

Table 2 shows the extent of climate exposure and vulnerability among respondents. These exposure patterns are consistent with broader urban climate trends in Dhaka, where recurrent seasonal heatwaves, annual monsoon flooding, and persistent waterlogging have become increasingly frequent due to rapid urban expansion, inadequate drainage infrastructure, and changing climatic conditions. A substantial proportion of households reported exposure to key climate stressors in the past 12 months, including heatwaves (70.6%) and flooding (64.1%). Prolonged waterlogging (>7 days) affected more than half of the sample (55.5%), indicating persistent environmental disruption. Climate-related impacts extend beyond environmental exposure to affect living conditions and livelihoods. Over half of respondents reported sanitation disruptions (55.7%) and income losses due to climate events (60.4%). Structural vulnerability is also evident. Poor housing materials (77.6%), overcrowding (67.4%), and inadequate ventilation (62.8%) are highly prevalent. Nearly half of households experienced disruptions in access to safe water during climate events, while 78.4% reported no access to formal disaster preparedness support. The composite Climate Vulnerability Index (CVI) indicates that 57.0% of households fall into the high vulnerability category, highlighting the cumulative and multidimensional nature of climate risk in informal settlements. These findings confirm that climate exposure in Dhaka is both widespread and structurally embedded.

**Table 2:** Extent of climate exposure and climate-induced vulnerability in informal urban settlements of Dhaka.

Climate exposure/vulnerability indicator	Operational definition	*n* (Yes)	%
Heatwave exposure (past 12 months)	Experienced ≥3 consecutive days of extreme heat affecting daily activities	271	70.6
Flood exposure (past 12 months)	Household affected by monsoon flooding at least once	246	64.1
Waterlogging duration >7 days	Standing water around dwelling lasting more than 1 week	213	55.5
Poor housing materials	Dwelling constructed with tin/bamboo/plastic sheets	298	77.6
Overcrowded housing	≥3 persons per room	259	67.4
Inadequate ventilation	No cross-ventilation or sealed indoor environment	241	62.8
Limited access to safe drinking water during climate events	Water source disrupted during floods/heatwaves	187	48.7
Sanitation disruption during flooding	Toilet unusable or contaminated during floods	214	55.7
Livelihood disruption due to climate events	Income loss due to heat/flood-related work interruption	232	60.4
No formal disaster preparedness support	No access to early warning, shelters, or local response services	301	78.4
Low adaptive capacity (composite index)	Low income + insecure housing + limited social support	219	57.0

Source: Field Survey, 2025.

### 4.3 Climate exposure and psychological stress

#### 4.3.1 Bivariate associations

Bivariate analysis reveals a consistent and statistically significant association between climate-related exposures and psychological stress (Table 3). The mean PSS-10 score for the sample is 19.8 (SD = 6.4), indicating moderate stress levels overall. Respondents exposed to climate stressors report significantly higher stress scores compared to those unexposed. Heatwave exposure is associated with a mean increase of 4.1 points in PSS-10 scores (21.4 vs. 17.3), while flood exposure corresponds to a difference of 4.9 points (22.0 vs. 17.1). Prolonged waterlogging (>7 days) shows an even larger difference of 5.7 points. Livelihood disruption due to climate events is associated with the highest stress levels (mea*n =* 23.1), suggesting that economic instability intensifies psychological burden. All observed differences are statistically significant (*p <* 0.001). These results indicate a strong and graded relationship between climate exposure and psychological stress.

**Table 3:** Association between climate-related exposures and psychological stress (PSS-10 scores) in informal urban settlements of Dhaka.

Climate exposure indicator	Category	Mean PSS-10 (SD)	Mean difference	*p*-value
Heatwave exposure (past 12 months)	Yes	21.4 (6.1)	+4.1	<0.001
	No	17.3 (5.8)	Ref	
Flood exposure (past 12 months)	Yes	22.0 (6.3)	+4.9	<0.001
	No	17.1 (5.6)	Ref	
Waterlogging duration	>7 days	22.6 (6.5)	+5.7	<0.001
	≤7 days/none	16.9 (5.4)	Ref	
Livelihood disruption due to climate events	Yes	23.1 (6.2)	+6.4	<0.001
	No	16.7 (5.3)	Ref	

#### 4.3.2 Multivariable regression analysis

Multivariable linear regression models (Table 4) confirm that climate-related exposures are significant predictors of psychological stress after adjusting for socioeconomic and health-related factors. In Model 1, heatwave exposure, flooding, and prolonged waterlogging are all independently associated with higher PSS-10 scores, explaining 29% of the variance. The inclusion of livelihood disruption in Model 2 increases explanatory power (Adjusted R^2^ = 0.37), with livelihood disruption emerging as a strong predictor. Model 3 further adjusts for demographic and health-related variables. Although the magnitude of climate exposure coefficients is attenuated, all remain statistically significant, indicating both direct and indirect pathways. Low household income, chronic illness, and female gender are also associated with higher stress levels. The final model explains 46% of the variance in psychological stress, demonstrating robust explanatory capacity. Overall, the findings suggest that climate exposure is an independent and significant predictor of psychological stress, even when accounting for socioeconomic vulnerabilities. Gender-disaggregated analysis further indicated that female respondents reported significantly higher psychological stress scores compared to males. In the fully adjusted model, female gender remained independently associated with elevated PSS-10 scores, suggesting heightened psychosocial vulnerability among women in climate-exposed informal settlements.

**Table 4:** Association between climate-related exposures and psychological stress (PSS-10 scores) in informal urban settlements of Dhaka.

Climate exposure indicator	Category	Mean PSS-10 (SD)	Mean difference	*p*-value
Heatwave exposure (past 12 months)	Yes	21.4 (6.1)	+4.1	<0.001
	No	17.3 (5.8)	Ref	
Flood exposure (past 12 months)	Yes	22.0 (6.3)	+4.9	<0.001
	No	17.1 (5.6)	Ref	
Waterlogging duration	>7 days	22.6 (6.5)	+5.7	<0.001
	≤7 days/none	16.9 (5.4)	Ref	
Livelihood disruption due to climate events	Yes	23.1 (6.2)	+6.4	<0.001
	No	16.7 (5.3)	Ref	

Taken together, these data points to one fact: there exist a clear and consistent connection between climate stressors and increased psychological stress. The size of the mean differences also indicates the possibility of a dose–response relationship with more disruptive or longer exposures generating bigger stress loads. These findings are empirical evidence of the hypothetical mechanism of climate vulnerability to negative health outcomes via psychosocial processes.

#### 4.3.3 A multivariable linear regression models

The multivariable linear regression models demonstrate a robust and graded association between climate-related exposures and perceived psychological stress (PSS-10 scores) among residents of informal urban settlements in Dhaka. In Model 1, which includes only environmental exposures, heatwave exposure, flood exposure, and prolonged waterlogging (>7 days) are all independently associated with significantly higher stress levels, explaining 29% of the variance in PSS-10 scores (see Table 5). Including livelihood disruption in Model 2 modestly attenuates the magnitude of the environmental coefficients, indicating partial mediation through economic pathways. Livelihood disruption emerges as a strong predictor (*β* = 4.01, *p <* 0.001), and overall model explanatory power increases substantially (Adjusted R^2^ = 0.37).

**Table 5:** Multivariable linear regression models.

Predictor variables	Model 1 *β* (SE)	Model 2 *β* (SE)	Model 3 *β* (SE)
Heatwave exposure	2.84*** (0.52)	2.41*** (0.50)	1.96*** (0.48)
Flood exposure	3.12*** (0.49)	2.77*** (0.47)	2.11*** (0.45)
Waterlogging >7 days	3.56*** (0.55)	3.08*** (0.52)	2.34*** (0.50)
Livelihood disruption	–	4.01*** (0.51)	3.27*** (0.48)
Female (ref: male)	–	–	1.42** (0.46)
Low household income	–	–	1.88*** (0.49)
Chronic illness	–	–	2.36*** (0.53)
Constant	15.6***	14.9***	13.7***

Model fit statistics. ***p < 0.001, **p < 0.01, and *p < 0.05.

### 4.4 Mediation analysis: climate vulnerability, psychological stress, and physical health

Mediation analysis (Table 6) demonstrates that psychological stress partially mediates the relationship between climate vulnerability and physical health outcomes. Climate vulnerability is significantly associated with increased likelihood of heat-related illness (OR = 2.41), infectious diseases (OR = 2.16), and chronic conditions (OR = 2.72). Climate vulnerability is also strongly associated with higher psychological stress (*β* = 4.12, *p <* 0.001), which in turn increases the risk of adverse health outcomes. The indirect (mediated) effects are statistically significant across all outcomes, with psychological stress accounting for 41.7% of the association with heat-related illness, 38.2% for infectious diseases, and 43.9% for chronic conditions. The persistence of significant direct effects alongside indirect effects indicates partial mediation. These findings suggest that psychological stress functions as an important psychosocial pathway linking climate vulnerability and adverse health outcomes; however, the observed relationships should be interpreted as complex and potentially bidirectional rather than strictly causal, particularly for heat-related and infectious diseases where direct environmental exposure remains central.

**Table 6:** Mediation analysis of psychological stress (PSS-10) in the relationship between climate vulnerability and physical health outcomes in Dhaka.

Physical health outcomes	Pathway	*β*/OR	95% CI	*p*-value
A. Heat-Related Illness	CVI → Heat-related illness (total effect)	OR = 2.41	1.58–3.67	<0.001
	CVI → PSS-10 (a path)	*β* = 4.12	3.31–4.93	<0.001
	PSS-10 → Heat-related illness (b path)	OR = 1.09	1.05–1.14	<0.001
	CVI → Heat-related illness (direct effect)	OR = 1.68	1.08–2.61	0.021
	Indirect (mediated) effect	OR = 1.43	1.19–1.72	<0.001
	Proportion mediated	41.7%	–	–
B. Infectious Diseases (Waterborne/Vector-borne)	CVI → Infectious disease (total effect)	OR = 2.16	1.44–3.23	<0.001
	CVI → PSS-10 (a path)	*β* = 4.12	3.31–4.93	<0.001
	PSS-10 → Infectious disease (b path)	OR = 1.07	1.03–1.11	<0.001
	CVI → Infectious disease (direct effect)	OR = 1.52	1.01–2.31	0.046
	Indirect (mediated) effect	OR = 1.31	1.12–1.56	<0.001
	Proportion mediated	38.2%	–	–
C. Chronic Health Conditions (Hypertension, Diabetes, Respiratory Disease)	CVI → Chronic condition (total effect)	OR = 2.72	1.74–4.26	<0.001
	CVI → PSS-10 (a path)	*β* = 4.12	3.31–4.93	<0.001
	PSS-10 → Chronic condition (b path)	OR = 1.11	1.06–1.16	<0.001
	CVI → Chronic condition (direct effect)	OR = 1.91	1.20–3.04	0.006
	Indirect (mediated) effect	OR = 1.53	1.25–1.89	<0.001
	Proportion mediated	43.9%	–	–

Mediator: Psychological stress (PSS-10, continuous). Exposure: Climate Vulnerability Index (CVI; high vs low/moderate). Outcomes: Binary health outcomes (yes = 1).

### 4.5 Predictors of adverse physical health outcomes

Binary logistic regression results (Table 7) identify key predictors of adverse physical health outcomes. The model demonstrates good fit (Nagelkerke R^2^ = 0.39; Hosmer–Lemeshow *p* = 0.62) and satisfactory classification accuracy (72.4%). Climate exposure variables are significant predictors of health risk. Each additional day of heatwave exposure increases the odds of illness by 9%, while flood and waterlogging exposures increase risk by 22 and 17%, respectively. Psychological stress is a strong independent predictor, with each unit increase in PSS-10 score associated with an 11% increase in the likelihood of adverse health outcomes. Socioeconomic factors show protective effects. Higher household income and greater educational attainment are associated with reduced health risk, while poor housing conditions significantly increase vulnerability. Female respondents exhibited significantly higher odds of adverse health outcomes, indicating gendered differences in climate-related vulnerability and psychosocial burden. These results underscore the combined influence of environmental exposure, psychological stress, and socioeconomic conditions on health outcomes.

**Table 7:** Binary logistic regression predicting adverse physical health outcome.

Predictor variables	Adjusted OR	95% CI	*p*-value
Climate exposure variables			
Heatwave exposure (days/year)	1.09	1.05–1.14	<0.001
Flood exposure (events/year)	1.22	1.10–1.35	<0.001
Waterlogging exposure (events/year)	1.17	1.07–1.28	0.001
Psychological stress			
PSS-10 score (per unit increase)	1.11	1.07–1.16	<0.001
Socioeconomic factors			
Household income (per 1,000 BDT increase)	0.92	0.88–0.96	<0.001
Education (years of schooling)	0.95	0.91–0.99	0.018
Housing type (Kutcha/Semi-pucca = 1)	1.46	1.01–2.11	0.044
Demographic controls			
Gender (Female = 1)	1.34	1.01–1.78	0.041
Age (years)	1.01	0.99–1.02	0.214

Model statistics: −2 Log Likelihood = 421.6. Nagelkerke R^2^ = 0.39. Hosmer–Lemeshow test: *χ*^2^ = 6.21, *p* = 0.62. Correct classification rate = 72.4%.

### 4.6 Structural pathways: climate exposure, stress, and health outcomes

**Figure 2:**
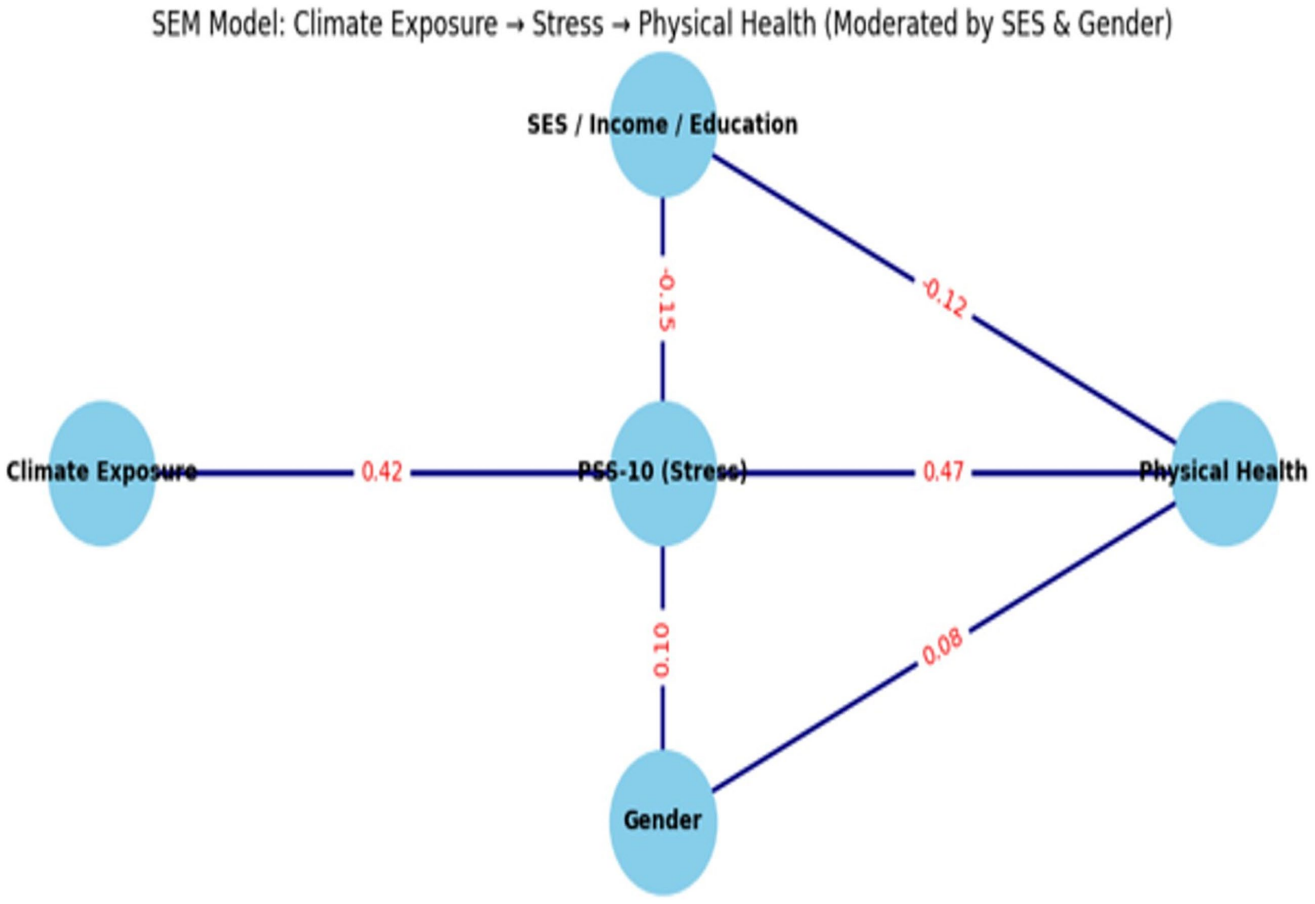
Structural equation model.

Structural Equation Modeling (SEM) results (Table 8) provide further evidence of the pathways linking climate exposure, psychological stress, and physical health. Climate exposure shows a significant positive association with psychological stress (*β* = 0.42, *p <* 0.001), while psychological stress significantly predicts poorer physical health (*β* = 0.47, *p <* 0.001). The total effect of climate exposure on health outcomes is also significant (*β* = 0.31, *p <* 0.001). The indirect (mediated) effect through psychological stress is substantial (*β* = 0.20, *p <* 0.001), indicating that a significant proportion of the exposure–health relationship operates through psychosocial mechanisms. The coexistence of direct and indirect effects confirms partial mediation, suggesting that climate exposure influences health both directly and through psychological stress pathways. Overall, the SEM results support a cumulative risk model in which environmental exposure increases psychological stress (as shown in Figure 2), which in turn contributes to adverse health outcomes.

**Table 8:** Mediation analysis: climate exposure → stress → physical health outcomes.

Pathway	Standardized *β*	SE	*p*-value	Effect type
Climate exposure → PSS-10	0.42	0.05	<0.001	Direct
PSS-10 → Physical health score	0.47	0.06	<0.001	Direct
Climate exposure → Physical health	0.31	0.07	<0.001	Total
Climate exposure → PSS-10 → Physical health	0.20	0.04	<0.001	Indirect/Mediated

Physical health score is a composite of heat-related illness, waterborne and vector-borne diseases, chronic condition exacerbation, and nutritional insecurity.

## 5 Discussion

The discussion directly interprets the principal findings presented in the Results section and links observed patterns of climate exposure, psychological stress, and health outcomes with relevant theoretical and empirical evidence to provide a coherent interpretation of the study findings.

### 5.1 Climate exposure as a chronic psychosocial stressor

This study examined the interrelationships between climate exposure, psychological stress, and health outcomes among low-income urban populations in Dhaka, providing empirical evidence of how recurrent environmental hazards translate into psychosocial and health risks. The findings demonstrate that climate-related exposures, particularly heatwaves, flooding, and prolonged waterlogging, are highly prevalent and systematically associated with elevated levels of perceived stress (PSS-10). These results are consistent with prior research indicating that environmental stressors are strongly linked to psychological strain in climate-vulnerable populations (7, 10, 11). However, while much of the existing literature emphasizes acute climate disasters, the present study highlights the significance of chronic and recurrent urban exposures. In line with emerging scholarship (8), the findings show that repeated exposure to heat stress and flooding generates sustained psychosocial burdens rather than short-term stress responses. By demonstrating measurable stress differentials across exposure groups, this study reinforces the conceptualization of climate exposure as a chronic psychosocial stressor, embedded in everyday living conditions within informal settlements. This extends the understanding of climate risk beyond episodic shocks to encompass cumulative and ongoing stress processes.

### 5.2 Psychological stress as a mediating mechanism

A central contribution of this study lies in identifying psychological stress as a key mechanism linking climate vulnerability to adverse physical health outcomes. The regression and mediation analyses consistently show that psychological stress partially mediates the relationship between climate exposure and health outcomes, while direct effects of exposure remain statistically significant. This pattern aligns with Stress Process Theory, which conceptualizes stress as a socially structured pathway through which environmental and structural stressors translate into health deterioration (23). The findings provide empirical support for this framework by demonstrating that climate-related stressors operate through both direct physiological pathways and indirect psychosocial mechanisms. At the same time, the results engage with ongoing debates in climate-health research regarding whether health impacts are primarily exposure-driven or resource-mediated (15). The evidence presented here suggests that these processes are not mutually exclusive. Rather, climate exposure initiates health risks, while socioeconomic disadvantage amplifies stress responses and exacerbates health outcomes. The observed partial mediation therefore supports integrated biopsychosocial frameworks, in which climate change affects health through interacting environmental, psychological, and social pathways (30). By quantifying the proportion of stress-mediated effects, this study advances empirical understanding of how psychosocial processes contribute to climate-related health risks.

### 5.3 Multidimensional vulnerability and social inequality

The findings further demonstrate that vulnerability in informal urban settlements is multidimensional, shaped by the intersection of environmental exposure, socioeconomic disadvantage, and demographic factors. Logistic regression results indicate that climate exposures, elevated stress levels, low income, limited education, and poor housing conditions significantly increase the likelihood of adverse health outcomes. These results are consistent with the Social Determinants of Health (SDH) framework, which emphasizes that health inequalities are embedded within broader structural and socioeconomic conditions (25). In this context, climate change acts as a compounding factor that intensifies existing inequalities, rather than operating as an isolated environmental risk. The higher risk observed among women reflects gendered and structurally mediated dimensions of climate vulnerability within informal urban settlements. Consistent with existing scholarship (34), women in low-income urban settings often face constrained access to adaptive resources, increased caregiving responsibilities, and heightened exposure to environmental stressors. These intersecting disadvantages contribute to disproportionate climate-health risks. The gender-disaggregated findings further support the argument that women experience disproportionate psychosocial and health burdens within climate-vulnerable urban environments.

Climate and health politics in the Global South, therefore, require a gender-responsive and intersectional policy perspective rather than a gender-neutral adaptation approach. The findings of this study suggest that women’s heightened psychosocial stress and adverse health risks are shaped not only by biological differences but also by socially structured inequalities, including unequal access to resources, insecure housing conditions, disproportionate caregiving responsibilities, limited decision-making power, and constrained adaptive capacity in informal urban settlements. These vulnerabilities are further intensified by poverty and infrastructural deprivation, indicating that climate-health risks are produced through intersecting social and structural inequalities rather than gender alone. In this context, gender-responsive climate-health governance should incorporate targeted psychosocial support, equitable access to healthcare and adaptation resources, women-centred disaster preparedness, and inclusive urban planning processes that recognize the differentiated experiences of climate vulnerability among women and marginalized populations in the Global South (34, 35). Rather than treating women solely as vulnerable populations, climate and health policy frameworks should also recognize women as critical agents in community resilience, local adaptation practices, and climate-health decision-making processes. Taken together, the findings indicate that climate vulnerability in Dhaka’s informal settlements is not solely environmental, but rather a layered phenomenon encompassing environmental, psychosocial, and structural dimensions. This underscores the importance of addressing climate-health risks through an equity-focused lens.

### 5.4 Implications for theory and climate–health research

This study contributes to climate-health scholarship in several important ways. First, it operationalizes climate vulnerability using an integrated exposure–sensitivity–adaptive capacity framework and empirically links it to psychological stress and health outcomes within a unified analytical structure. Second, the study extends Stress Process Theory by positioning climate exposure as a chronic structural stressor embedded within urban inequality. This conceptualization moves beyond traditional applications of the theory by incorporating environmental change as a persistent source of stress proliferation. Third, through the application of mediation analysis and Structural Equation Modeling (SEM), the study quantifies the extent to which psychological stress mediates climate-health relationships an approach that remains limited in low- and middle-income urban contexts. By integrating theoretical frameworks with advanced quantitative methods, the study strengthens pathway-based approaches in climate epidemiology and enhances theoretical–methodological alignment in Global South research settings.

### 5.5 Policy and practice implications

The findings have significant implications for climate adaptation and public health policy in low-income urban contexts. First, climate adaptation strategies must prioritize informal settlements, where exposure to environmental hazards is most concentrated. Structural interventions—such as improved drainage systems, flood-resilient housing, and access to safe water—are essential for reducing direct exposure to climate risks. Second, identifying psychological stress as a mediating factor underscores the need to integrate mental health services into climate adaptation programs. This includes community-based counseling, stress management interventions, and psychosocial support, particularly during and after extreme climate events. Health systems should be adapted to recognize and respond to stress-related health risks in climate-vulnerable populations. Third, policies must address underlying socioeconomic and gender inequalities that amplify climate-health risks. Interventions to improve income security, education, housing conditions, and access to services are critical for strengthening adaptive capacity. Special attention should be given to women and other marginalized groups to ensure equitable access to resources and support systems. Finally, the development of integrated climate–health surveillance systems is essential for evidence-based policymaking. Monitoring environmental exposures alongside psychological and health outcomes can support early identification of risks, targeted interventions, and evaluation of policy effectiveness. Such integrated approaches are necessary to build resilience in rapidly urbanizing and climate-vulnerable settings like Dhaka.

#### 5.5.1 Implications for public policy, international climate diplomacy, and cross-sectoral action in the Global South

The findings of this study have broader implications for climate change and health policy across rapidly urbanizing regions of the Global South, where informal settlements experience disproportionate exposure to recurrent climate hazards alongside structural socioeconomic inequalities. The results indicate that climate-related health risks cannot be addressed solely through disaster-response or infrastructure-centered adaptation strategies. Instead, climate policy frameworks in the Global South should integrate urban public health, mental health services, housing policy, water and sanitation systems, and social protection mechanisms within a coordinated climate-health governance approach. Given the significant mediating role of psychological stress identified in this study, climate adaptation policies should explicitly incorporate psychosocial and mental health support as essential components of climate resilience planning rather than treating mental health as a secondary or isolated concern (8, 15).

The findings further suggest the need for climate-sensitive urban health policies that prioritize informal settlements through investments in flood-resilient housing, heat mitigation infrastructure, drainage improvement, safe water access, early warning systems, and community-based health preparedness programs. Social protection measures, including livelihood support, emergency cash assistance, and targeted support for women and other socially vulnerable populations, are also critical for reducing cumulative climate-health vulnerability. These interventions are particularly important in Global South cities where rapid urbanization, infrastructural inequality, and weak adaptive capacity intensify climate-related health risks.

At the international level, the study highlights several priorities for climate diplomacy and global climate governance. First, international climate negotiations should place greater emphasis on urban health vulnerability, psychosocial health impacts, and climate-related mental health burdens within adaptation and loss-and-damage agendas. Second, climate financing mechanisms should allocate greater resources toward locally driven urban adaptation and climate-health programs in low- and middle-income countries, particularly for informal settlements that face chronic underinvestment. Third, international climate diplomacy should promote equitable climate governance by recognizing that climate-health risks in the Global South are deeply linked to historical inequalities in global emissions, uneven urban development, and unequal adaptive capacity. Strengthening global cooperation on climate-resilient public health systems, urban infrastructure, and climate-health surveillance will therefore be essential for reducing health inequities under conditions of accelerating climate change (4, 35).

Finally, the study underscores the importance of cross-sectoral action involving public health agencies, urban planning authorities, environmental institutions, social protection systems, and local governance structures. Because climate-related health risks emerge through interconnected environmental, psychosocial, and socioeconomic pathways, isolated sectoral interventions are unlikely to be sufficient. Integrated climate-health governance frameworks that combine environmental adaptation, mental health support, urban infrastructure improvement, and social equity measures are necessary to strengthen resilience in vulnerable urban communities across the Global South (25).

### 5.6 Limitations and directions for future research

Several limitations should be considered when interpreting the findings. First, the cross-sectional design limits the ability to establish causal relationships among climate exposure, psychological stress, and health outcomes. Although mediation analysis provides insight into potential pathways, longitudinal data are required to confirm temporal dynamics. Second, the reliance on self-reported measures may introduce recall and reporting bias, particularly for climate exposure and health outcomes. Future studies should incorporate objective environmental and physiological measures to enhance measurement validity. Third, the study focuses on informal settlements in Dhaka, which may limit the generalizability of findings to other urban or rural contexts. Comparative studies across different cities or regions would help to identify context-specific and universal patterns of climate vulnerability. Finally, although key socioeconomic variables were included, other potentially relevant factors, such as social networks, pre-existing mental health conditions, and neighborhood-level infrastructure, were not fully captured. Future research should adopt more comprehensive, longitudinal designs to better understand the complex, dynamic nature of climate-health relationships.

## 6 Conclusion

This study demonstrates that climate-induced exposures particularly heatwaves, flooding, and prolonged waterlogging are pervasive in low-income urban settlements in Dhaka and are significantly associated with elevated psychological stress and adverse physical health outcomes. By integrating environmental, psychosocial, and health dimensions within a single analytical framework, the findings provide robust empirical evidence that climate vulnerability operates through both direct and indirect pathways. A key contribution of the study is the identification of psychological stress as a partial mediating mechanism linking climate exposure to physical health risks. This highlights that climate change is not only an environmental hazard but also a psychosocial stressor that shapes health outcomes through complex, multidimensional processes. The persistence of direct effects alongside mediated pathways further suggests that climate-related health risks are both physiologically and socially embedded.

The study also underscores the role of structural inequalities in shaping climate vulnerability. Socioeconomic disadvantage, poor housing conditions, and gendered disparities significantly amplify both exposure and health risks, reinforcing the importance of viewing climate change through a social determinants of health perspective. In this context, vulnerability in informal urban settlements emerges as a layered phenomenon, where environmental hazards intersect with psychosocial stress and structural disadvantage to produce cumulative health burdens. From a policy perspective, the findings emphasize the need for integrated and context-sensitive interventions that simultaneously address environmental exposure, mental health, and socioeconomic vulnerability. Climate adaptation strategies in rapidly urbanizing settings such as Dhaka must extend beyond infrastructure to include psychosocial support systems and equity-focused public health approaches. Overall, this study contributes to advancing climate–health research in the Global South by providing a pathway-based understanding of how climate vulnerability translates into health outcomes. By empirically demonstrating the interconnected roles of environmental exposure, psychological stress, and structural inequality, it highlights the necessity of multidimensional and interdisciplinary approaches to addressing the health impacts of climate change in vulnerable urban populations.

## Data Availability

The raw data supporting the conclusions of this article will be made available by the authors, without undue reservation.
